# Supplementary material for: Transcriptomic signatures of brain regional vulnerability to Parkinson’s disease
Source: Commun Biol. 2020 Mar 5;3:101. doi: 10.1038/s42003-020-0804-9 (PMC7058608; doi:10.1038/s42003-020-0804-9)
Supplement: Supplementary file 1 — Supplementary Information [file 42003_2020_804_MOESM1_ESM.pdf]

Supplementary Figures

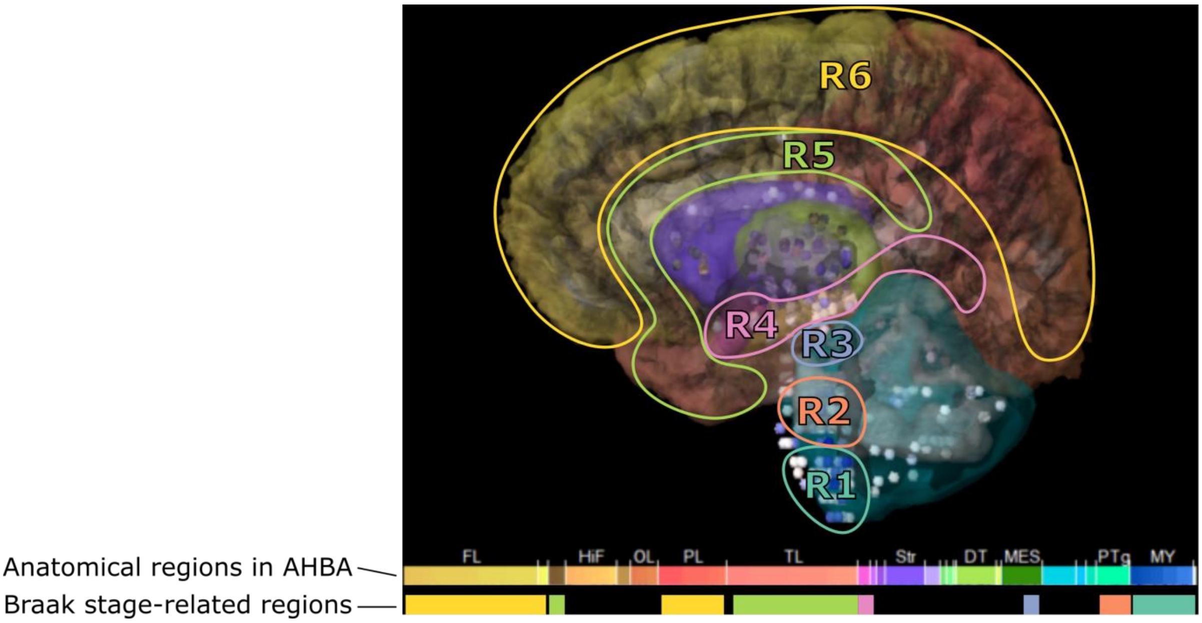

Supplementary Fig. 1 Defining Braak stage-related regions in the Allen Human Brain Atlas (AHBA). Top row shows the anatomical regions and annotated colors as defined in the AHBA (image credit: Allen Institute). Bottom row shows regions that are assigned as Braak stage-related regions where colors correspond to each one of the six regions R1-R6. See Supplementary Table 1 for names of anatomical structures and sample size information per AHBA donor.

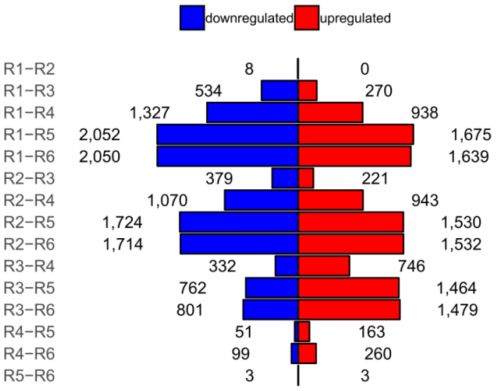

Supplementary Fig. 2 Number of differentially expressed genes between all pairs of six Braak stage-related regions R1-R6. Differentially expressed genes were downregulated (blue) or upregulated (red) in the second region compared to the first region. Respectively, this supports a negative or positive correlation with Braak stages.

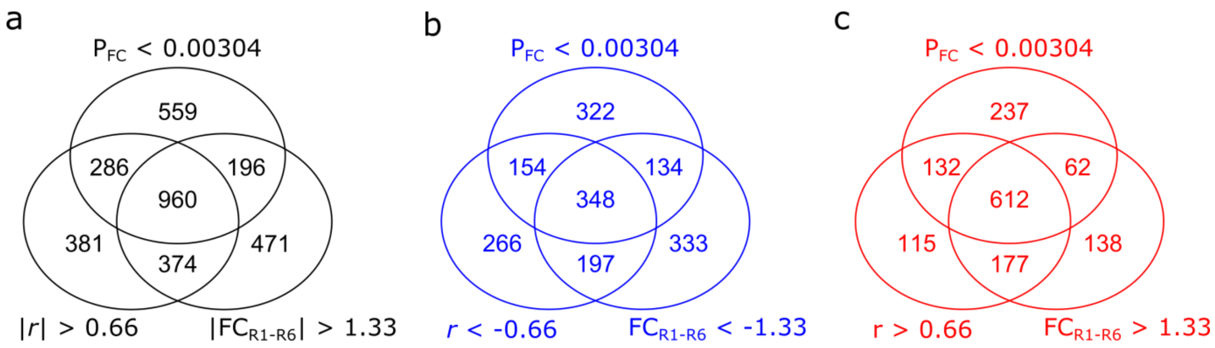

Supplementary Fig. 3 Venn diagrams showing the selection of Braak stage-related genes (BRGs). BRGs were selected based on (i) the highest absolute Braak label correlation (|*r*|), (ii) highest absolute FC between R1 and R6 ($|FC_{R1\text{-}R6}|$), and (iii) smallest BH-corrected P-values of the FC (P_{FC}; two-sided t-test). (a) The top 10% (2,001) ranked genes for each criterium resulted in 960 BRGs. Differentiating between two expression patterns shows there are (b) 348 negatively and (c) 612 positively correlated BRGs.

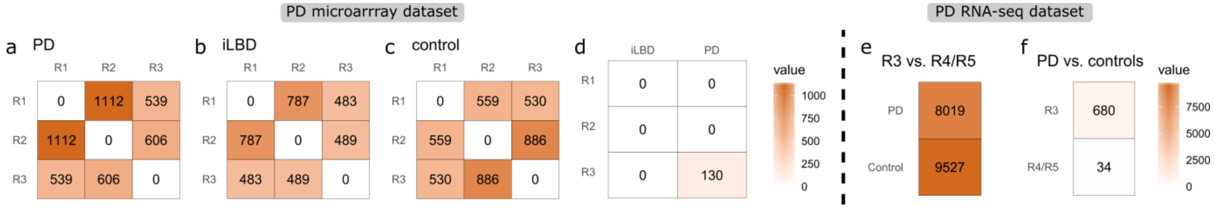

Supplementary Fig. 4 Number of differentially expressed genes in PD microarray and RNA-sequencing datasets. Differential expression (t-test) was analyzed (a,b,c,e) between regions within groups of individuals and (d, f) between PD or iBLD patients vs. non-demented age-matched controls within regions. Results showed that the expression variation is greater between regions than between groups of individuals.

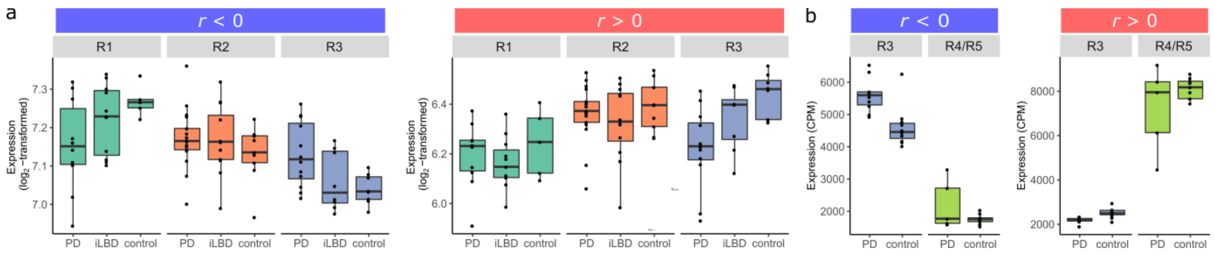

Supplementary Fig. 5 Expression of BRGs in groups of individuals per region in the PD datasets. Patterns across regions are replicated across disease conditions for (a) the PD microarray and (b) PD RNA-seq datasets. Same data is shown in Fig. 2f and Fig. 2g where samples are plotted per region within groups of individuals (number of samples in Supplementary Table 2 and 7). The boxplots indicate the median and interquartile range (25th and 75th percentiles) with whiskers indicating 1.5 times the interquartile range; outliers beyond the whiskers are plotted individually.

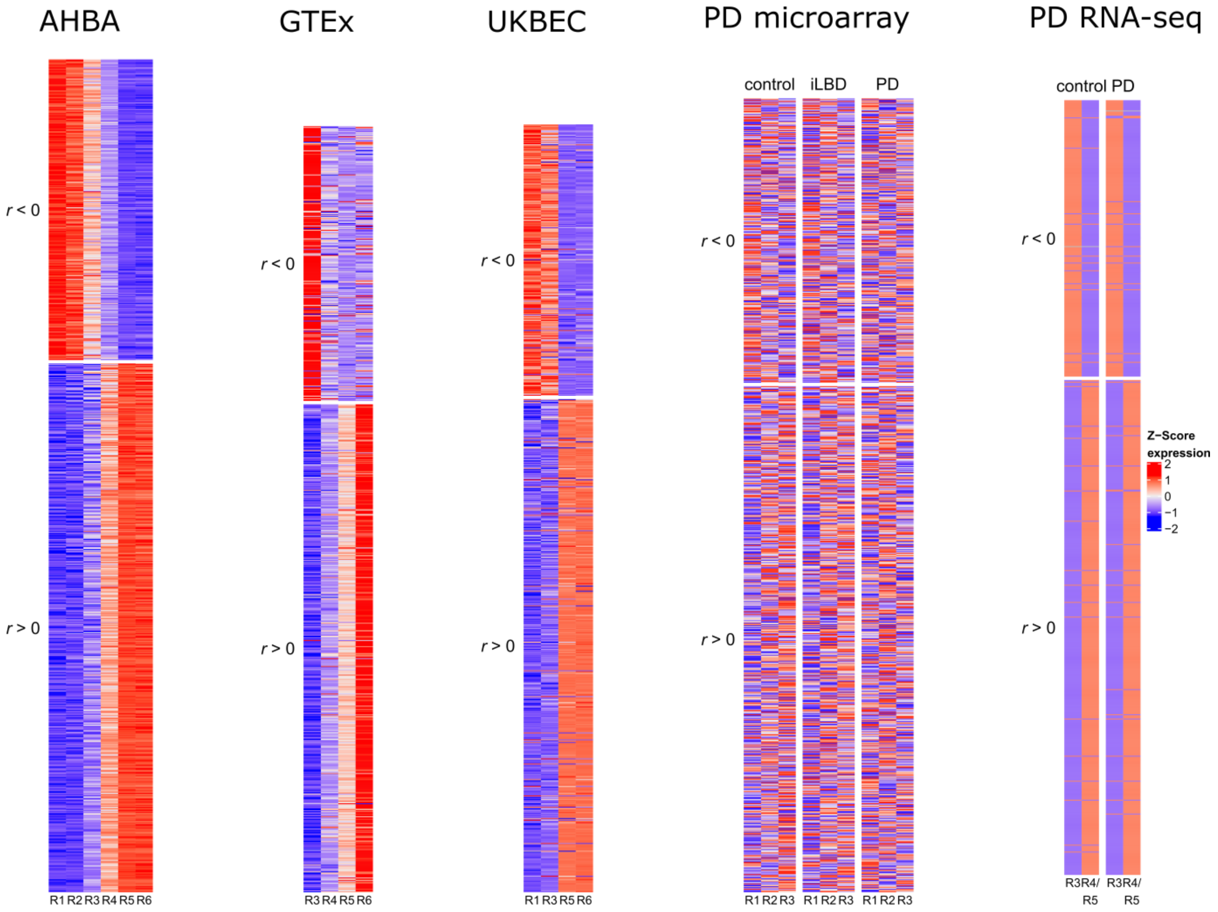

Supplementary Fig. 6 Expression of individual BRGs in AHBA and validated datasets GTEx, UKBEC, PD microarray, and PD RNA-seq.

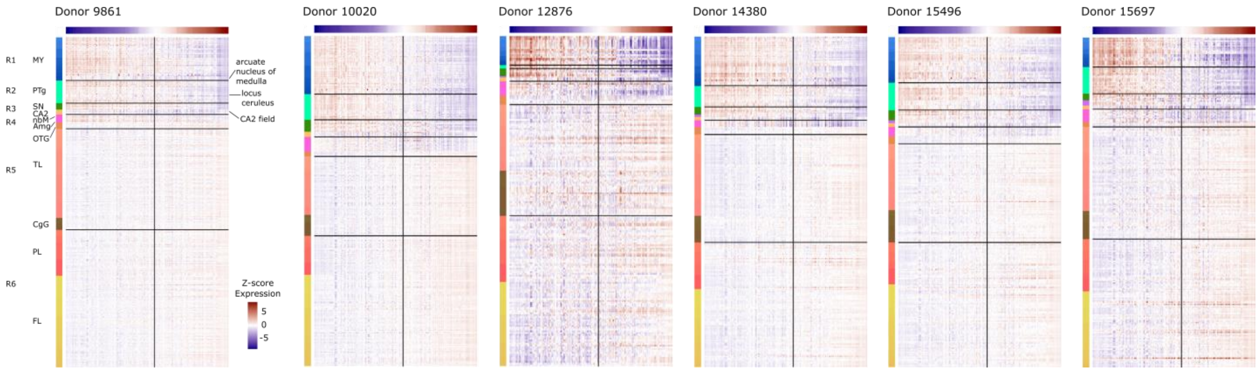

Supplementary Fig. 7 Expression heatmap module eigengenes across Braak region 1-6 for all six donors. Modules were obtained by hierarchical clustering of genes co-expressed in Braak stage-related regions R1-R6 (rows) with anatomical structures being colored. Eigengenes were sorted based on their correlation with Braak labels (columns). Expression of highly correlated eigengenes either increases from high (red) to low (blue) expression or decreases across regions R1-R6. All modules showed more extreme values (high and low) in R1-R3 compared to R4-R6.

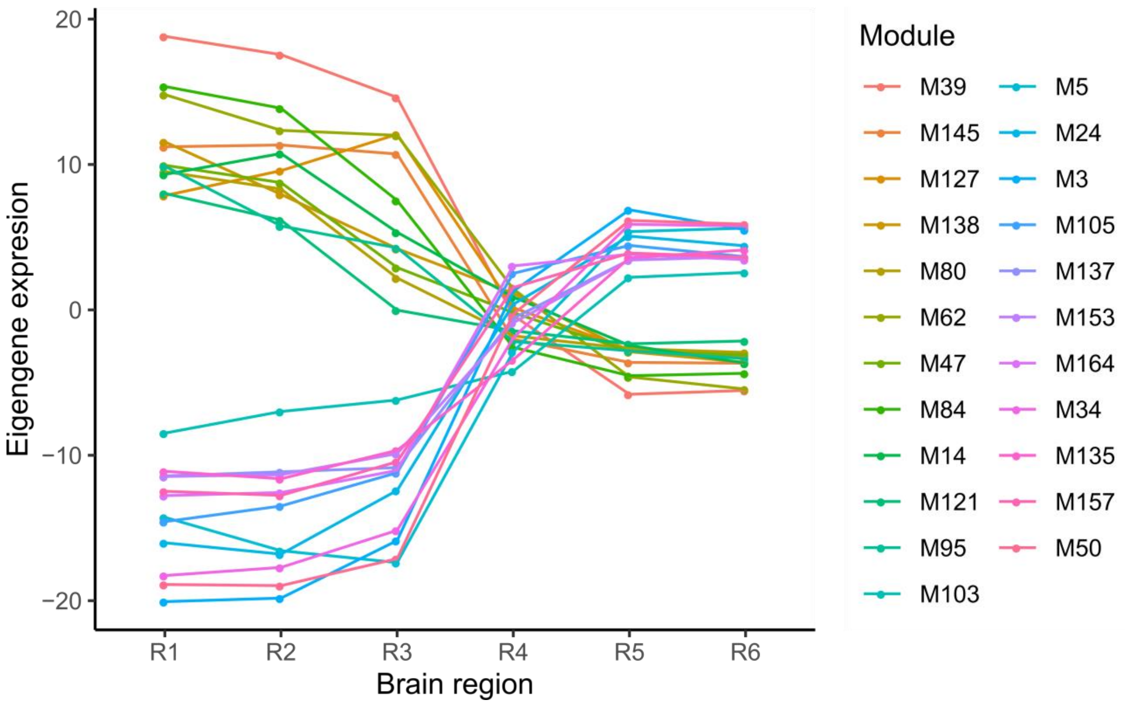

Supplementary Fig. 8 Eigengene expression of 23 modules correlated with Braak stages. Plot shows the mean expression across the six donors.

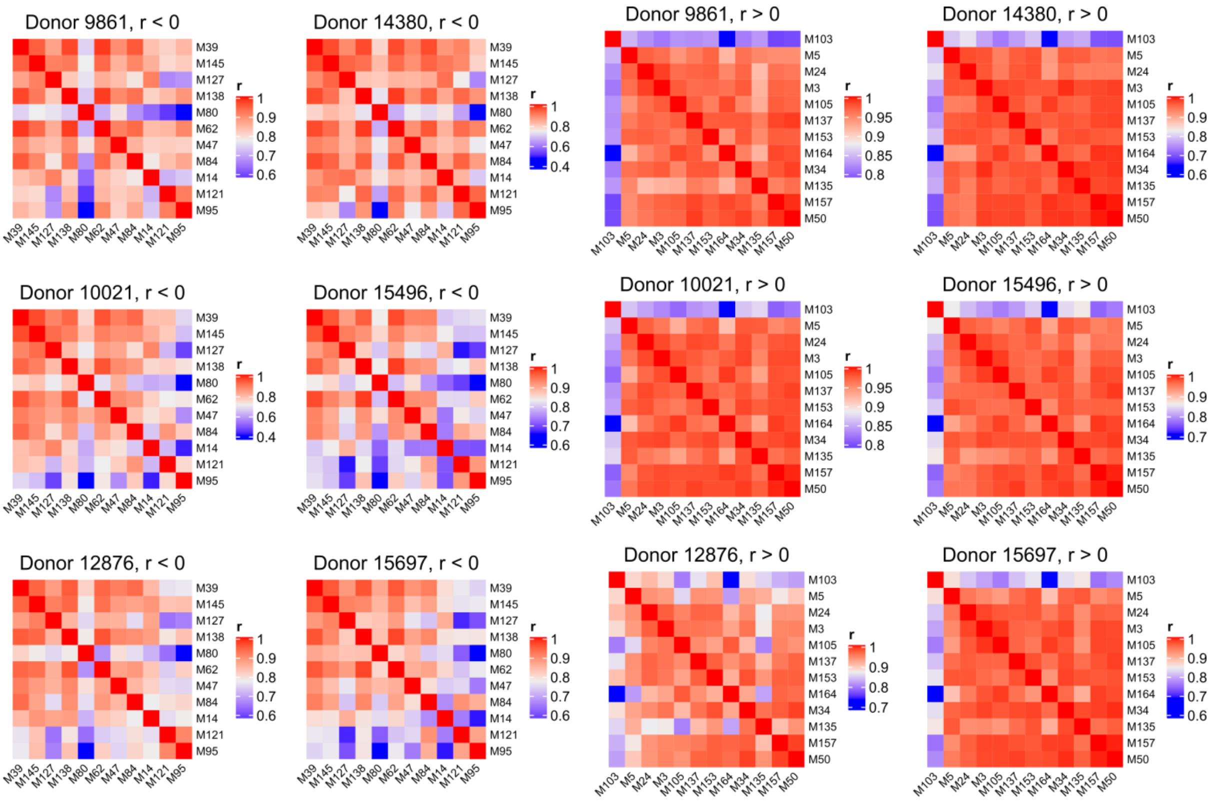

Supplementary Fig. 9 Pearson correlation between modules that were negatively ($r < 0$) or positively ($r > 0$) correlated with Braak stages. Among 167 modules, we found 23 modules showing a significant correlation with Braak stage labels. Here, we plot the correlation between module eigengenes for those that were either negatively or positively correlated and for each one of the six donors in the Allen Human Brain Atlas. Mostly the negatively correlated modules showed distinct expression patterns.

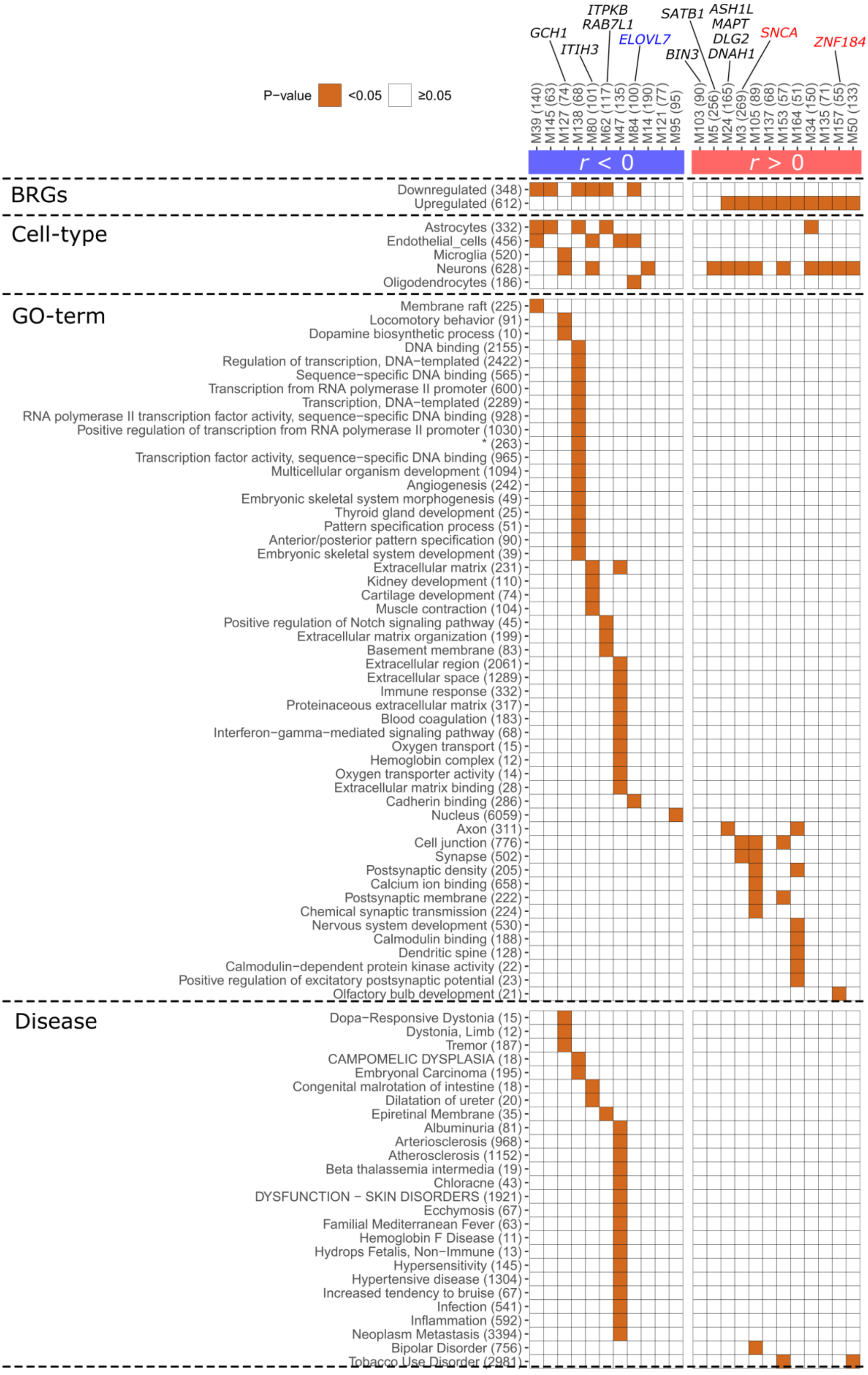

* Transcriptional activator activity, RNA polymerase II core promoter proximal region sequence−specific binding

Supplementary Fig. 10 Full table for module enrichment of Braak stage related genes (BRGs), cell-types, GO-terms, and disease-associated genes. This table shows all the significant associations we found for 23 Braak stage related modules. A more concise version of this table showing associations relevant to PD is presented in Fig. 3c.

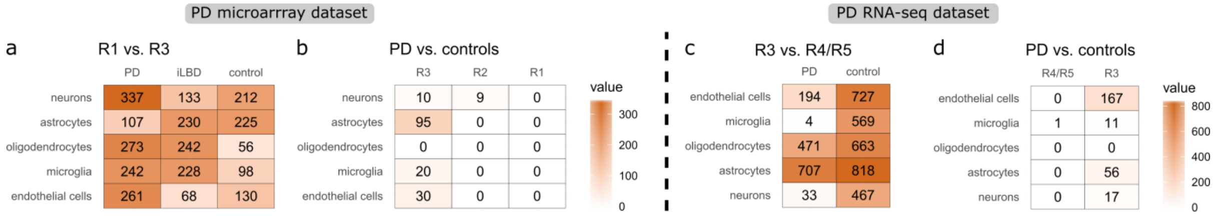

Supplementary Fig. 11 Number of differentially expressed BRGs in the PD datasets after correction for cell-type composition. Differential expression after cell-type correction with PSEA was analyzed (a, c) between regions within groups of individuals and (b, d) between PD patients vs. non-demented age-matched controls within regions. Results showed that the expression variation is greater between regions than between groups of individuals, even after correcting expression differences for five main cell-types.

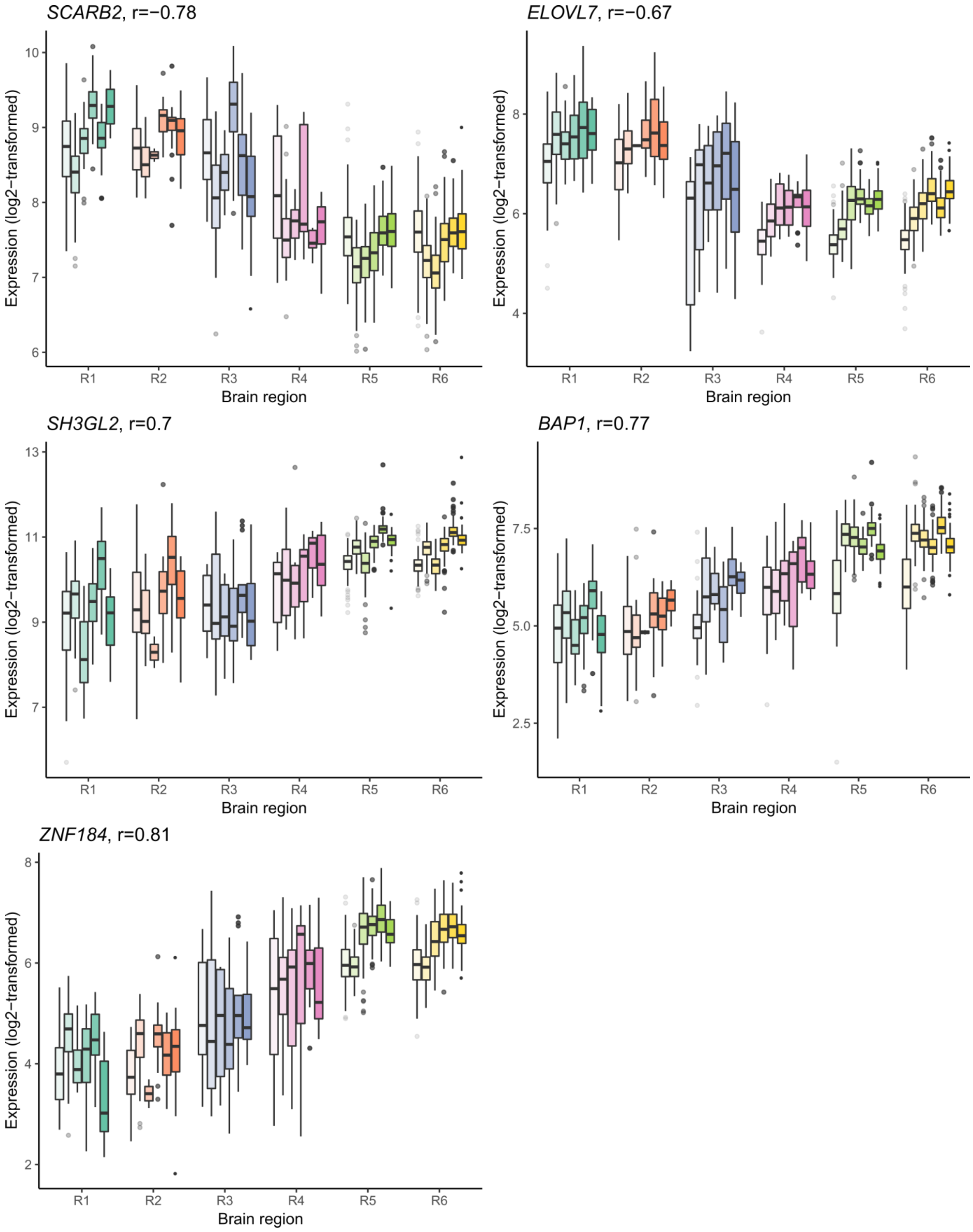

Supplementary Fig. 12 Physiological expression of PD-variant associated genes across regions involved in Braak Lewy body stages. *SCARB2*, *ELOVL7*, *SH3GL2*, *BAP1*, and *ZNF184* have been associated to PD in gene-wide association studies. Here, we identified expression patterns across regions involved in Braak Lewy body stages of PD (number of samples in Supplementary Table 1). The boxplots indicate the median and interquartile range (25th and 75th percentiles) with whiskers indicating 1.5 times the interquartile range; outliers beyond the whiskers are plotted individually.

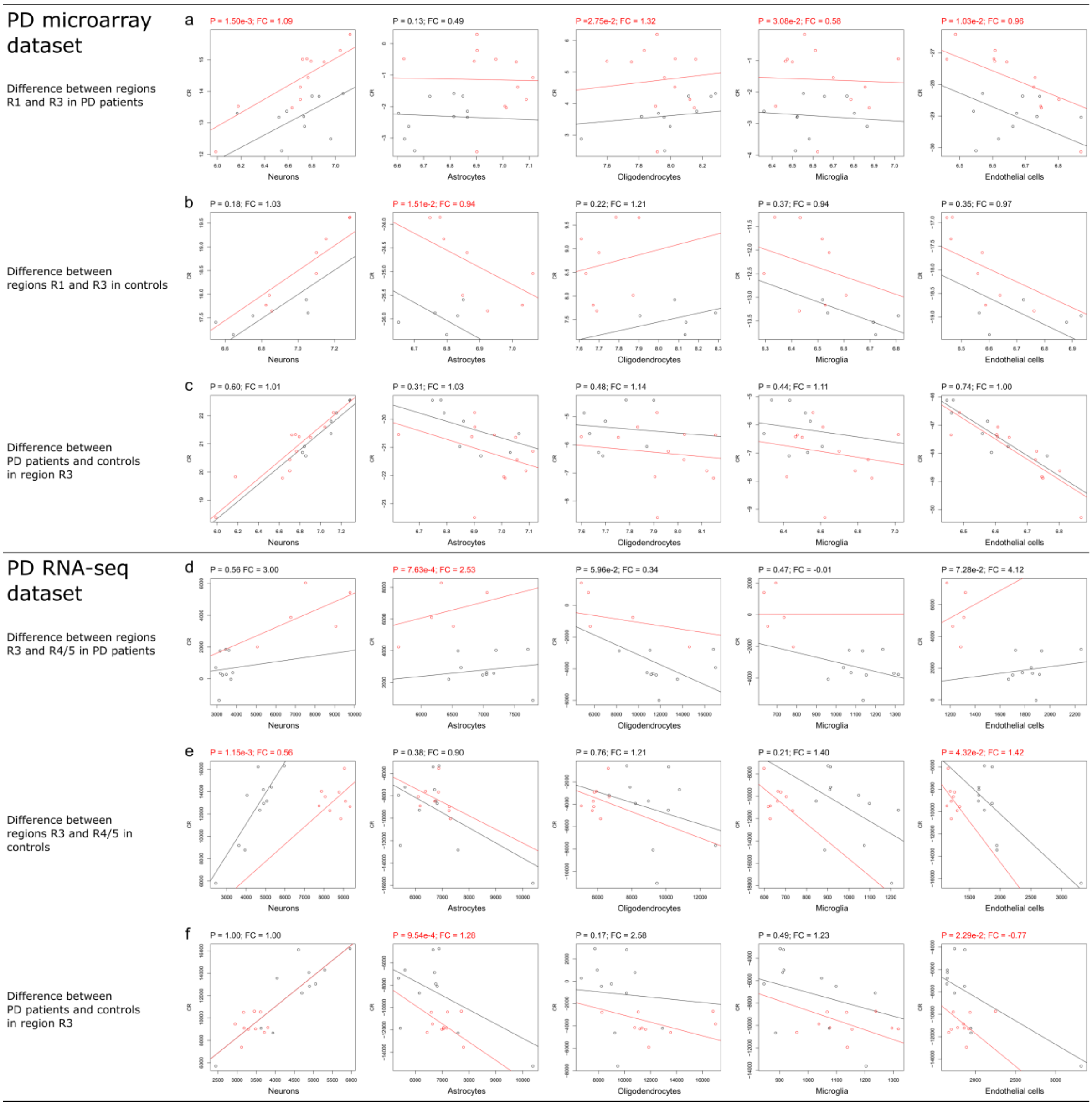

Supplementary Fig. 13 Differential expression of *SNCA* in PD cohorts corrected for cell-type abundance with PSEA. A microarray and RNA-sequencing dataset of PD cohorts were analyzed for differential expression while correcting for the abundance of neurons, astrocytes, oligodendrocytes, microglia, and endothelial cells based on the average expression of cell-type markers. PSEA analysis was applied between regions as well as between conditions PD and control. Significant BH-corrected P-values are highlighted in red text together with cell-type specific fold-changes (FC; slope change of red line). (a) In microarray data of PD patients, *SNCA* was still significantly differentially expressed between regions R1 (black) and R3 (red) after correcting for neurons, oligodendrocytes, microglia, and endothelial cells. (b) In age-matched controls, *SNCA* was only significantly differentially expressed between R1 and R3 when the change was specific to astrocytes. (c) *SNCA* was not significantly differential expressed between controls (red) and PD patients (black) within region R3 given any of the cell-types. (d) In RNA-seq data of PD patients, *SNCA* was differentially expressed between regions R3 (black) and R4/5 (red) after correcting for astrocytes. (e) In age-matched controls, *SNCA* was differentially expressed between regions R3 (black) and R4/5 (red) after correcting for neurons and endothelial cells. (f) When comparing samples from region R3 between PD patients (red) and controls (black), *SNCA* was only significant given astrocytes and endothelial cells.

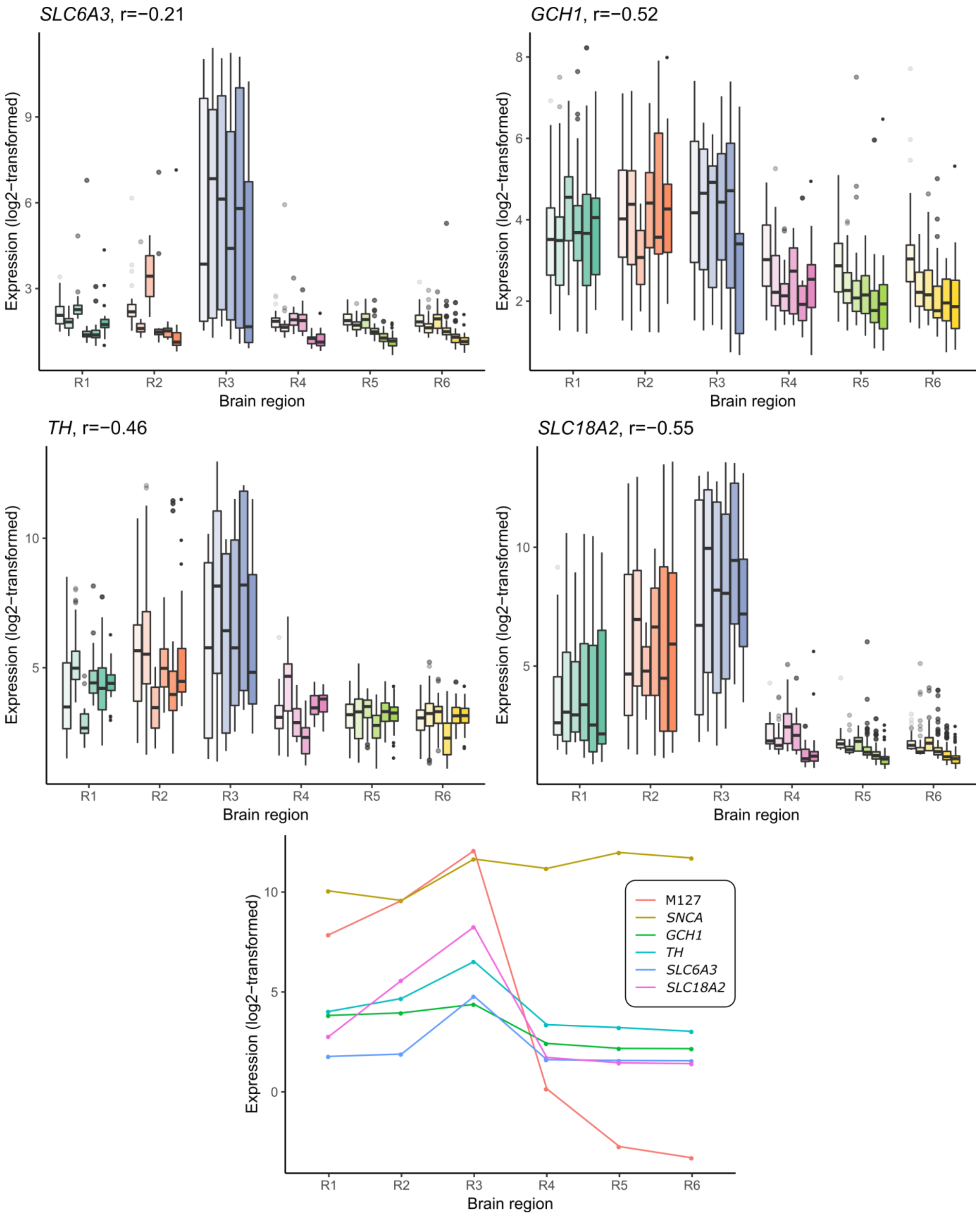

Supplementary Fig. 14 Physiological expression of dopaminergic genes across regions involved in Braak Lewy body stages. *SLC6A3* (*DAT*), *GCH1*, *TH*, and *SLC18A2* (*VMAT2*) were co-expressed in module M127 which was functionally related to dopamine synthesis. Bottom plot shows the mean of medians across the six donors. The boxplots indicate the median and interquartile range (25th and 75th percentiles) with whiskers indicating 1.5 times the interquartile range; outliers beyond the whiskers are plotted individually.

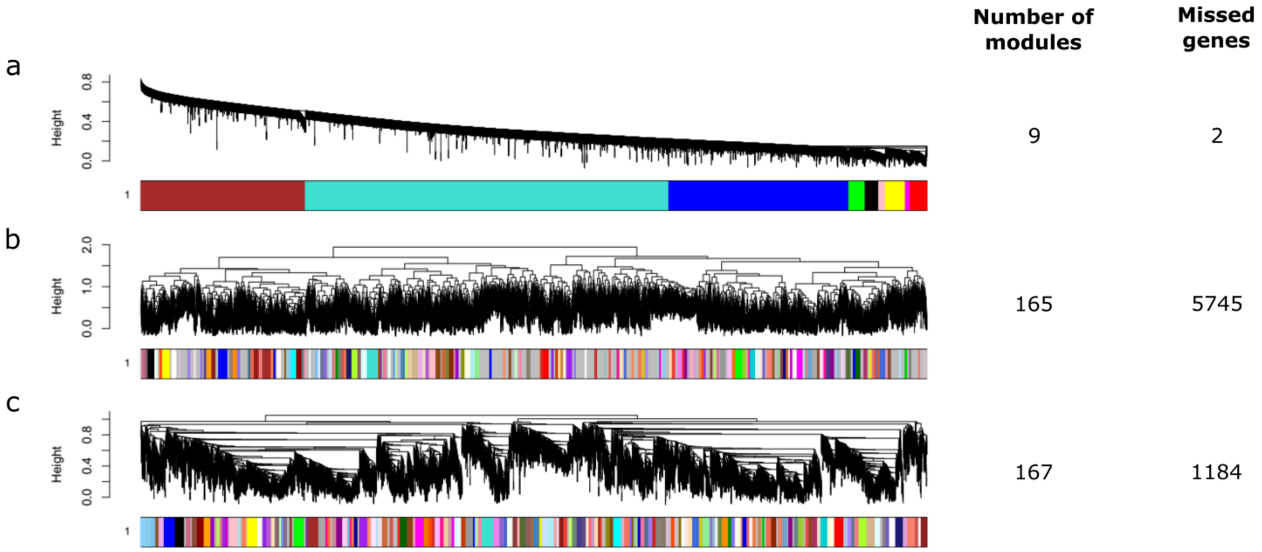

Supplementary Fig. 15 Hierarchical clustering based on co-expression of genes in Braak regions 1 to 6. Dendrograms and colored modules obtained with (a) single, (b) complete and (c) average linkage are shown together with the number of missed genes and number of modules.

Supplementary Tables

Supplementary Table 1 Number of samples for each Braak stage-related region across the six donors in AHBA. Each one of the Braak regions is represented by one or multiple anatomical structures; names are according to the ontology of AHBA.

**Braak stage-related regions**	**Anatomical structures**	**Donor 9861**	**Donor 10021**	**Donor 12876**	**Donor 14380**	**Donor 15496**	**Donor 15697**	**Total**
R1	Myelencephalon	76	85	16	44	35	23	279
R2	Pontine tegmentum	40	38	2	19	21	21	141
R3	Substantia nigra, basal nucleus of meynert, right, basal nucleus of meynert, left, CA2 field	20	25	7	12	13	12	89
R4	Amygdala, occipito-temporal gyrus	25	29	13	13	13	14	107
R5	Cingulate gyrus, temporal lobe	178	117	62	97	76	88	618
R6	Frontal lobe, parietal lobe	242	192	84	112	96	101	827

Supplementary Table 2 Number of samples in PD microarray dataset. Braak stage-related regions are represented by one anatomical structure.

**Braak stage-related regions**	**Anatomical structures**	**PD**	**iLBD**	**Controls**
R1	Medulla oblongata (Myelencephalon)	10	11	5
R2	Locus ceruleus	17	12	9
R3	Substantia nigra	12	8	8

Supplementary Table 3 Number of samples in PD RNA-seq dataset. Braak stage-related regions are represented by one anatomical structure.

**Braak stage-related regions**	**Anatomical structures**	**PD**	**Controls**
R3	Substantia nigra	10	11
R4/R5	Medial temporal gyrus	5	9

Supplementary Table 4 Literature overview on protective and toxic effects of α-synuclein. Laboratory studies that examined the effect of wild-type or mutant α-synuclein using various experiments. A53T and A30P are familial Parkinson's disease pathogenic mutations. DA: dopamine, PD: Parkinson's disease.

**Study**	**Samples**	**Molecular type of α-synuclein**	**Outcome**	**Wild-type α-synuclein is protective?**
Neystat et al. (1999)[1]	Substantia nigra and cortex from 9 PD patients and 8 controls	mRNA	Less α-synuclein mRNA was observed in the substantia nigra of PD patients implying that decreased levels may play a role in the pathogenesis. It is unlikely that this decrease simply reflects neuronal loss because a similar difference was observed after normalizing for *VMAT2* (a dopaminergic neuronal marker) expression.	Yes
Kholodilov et al. (1999)[2]	Rat model of induced programmed cell death in substantia nigra (striatal lesions with quinolinic acid)	Protein, mRNA, DNA	α-Synuclein does not play in direct role in mediating cell death, but may play a role in compensatory or plasticity response in surviving neurons.	Yes

	and mesencephalic DA neurons.			
Abeliovich et al. (2000)[3]	Brains of wild-type and α-synuclein -/- mice.	Protein	Mutant mice display altered DA release, and reduced striatal DA and amphetamine-stimulated locomotion. Thus, α-synuclein is an essential presynaptic, activity-dependent negative regulator of DA neurotransmission.	Yes
Alves da Costa et al. (2000)[4]	Mouse neocortical neuronal cell line overexpressing wild-type or A53T mutant α-synuclein.	Protein	Wild-type α-synuclein exerts an antiapoptotic effect in neurons that appears to be abolished by the PD-related mutation.	Yes
Alves da Costa et al. (2002)[5]	Mouse neocortical neuronal cell line overexpressing wild-type α-synuclein.	Protein	α-Synuclein lowers the p53-dependent caspase 3 activation and natural toxin 6-hydroxydopamine abolishes the antiapoptotic phenotype by triggering α-synuclein aggregation.	Yes
Lee et al. (2001)[6]	Human teratocarcinoma and neuroblastoma cell lines overexpressing wild-type, A30P and A53T mutant α-synuclein.	Protein	Mutant α-synucleins increased levels of 8-hydroxyguanine, protein carbonyls, lipid peroxidation and 3-nitrotyrosine, and markedly accelerated cell death in response to all the insults examined. Wild-type α-synuclein is cell line- and insult-specific, suggesting both protective and non-protective properties.	Yes
Stefanis et al. (2001)[7]	Rat pheochromocytoma (dopaminergic) PC12 cell line overexpressing wild-type and A53T mutant α-synuclein.	Protein	Mutant α-synuclein enhances cell death, induces accumulation of autophagic–vesicular structures, and results in a loss of catecholamine storage granules and the capacity for depolarization-induced DA release. These effects may involve lysosomal and proteasomal degradation systems.	Yes
Hashimoto et al. (2002)[8]	Mouse hypothalamic tumor neuronal cell line overexpresssing α-synuclein, β-synuclein or control vector.	Protein, mRNA	Increased α-synuclein expression might protect cells from oxidative stress by inactivation of JNK via increased expression of JIP-1b/IB1.	Yes
Seo et al. (2002)[9]	Rat primary cortical and hippocampal neurons and microglia, and various neuronal cell lines.	Protein	At low concentrations, α-synuclein protects neurons against stress conditions through the PI3/Akt signaling pathway. At overexpression α-synuclein induced cytotoxicity.	Yes and no
Cabin et al. (2002)[10]	Whole brain and hippocampal neurons from α-synuclein -/- and +/+ mice	Protein	α-Synuclein is required for the genesis, localization, and/or maintenance of at least some subset of vesicles that make up the reserve or resting pools of presynaptic vesicles	Yes
Wersinger et al. (2003)[11]	Ltk- fibroblasts, human neuroblastoma-derived SK-N-MC cells, and HEK 293 human embryonic kidney cells transfected with DAT and α-synuclein, and primary rat mesencephalic cultures.	Protein	α-Synuclein forms a complex with DAT in both rat brain tissues and transfected cells, which regulates the amount of DAT at the plasma membrane. In basal situations, α-synuclein tends to markedly decrease DA uptake by DAT as well as decrease DA-mediated oxidative stress and cell death. The primary function of presynaptic α-synuclein in dopaminergic neurons may be the regulation of DA homeostasis	Yes
Zourlidou et al. (2003)[12]	Neuronal ND7 cell line overexpressing wild-type, A30P and A53T mutant α-synuclein.	protein	Mutations in α-synuclein convert the protein from one that is able to modulate cell death into a protein with a damaging effect in neuronal cells.	Yes
Kingsbury et al. (2004)[13]	human substantia nigra and cortex of PD and control subjects.	mRNA	In substantia nigra neurons there seemed to be a negative correlation between cellular mRNA expression and disease duration. Lewy body formation is unlikely to be the result of overexpression of α-synuclein.	Yes
Chandra et al. (2004)[14]	α -/- β-/- and α -/- β+/+ synuclein mice.	Protein	Synucleins are not essential components of the basic machinery for neurotransmitter release but may contribute to the long-term regulation and or maintenance of presynaptic function.	Yes
Chandra et al. (2005)[15]	Mice that overexpress wildtype human α-synuclein, A30P mutant human α-synuclein, A53T mutant human α-synuclein, or wild-type mouse α-synuclein.	Protein	Transgenic expression of α-synuclein unexpectedly abolishes the lethal phenotype created by deletion of presynaptic chaperone protein CSPα in mice.	Yes

Oksman et al. (2006)[16]	α-Synuclein -/- and +/+ mice.		A lack of α-synuclein sensitized the brain reward system, implying that the levels of α-synuclein expression may predispose an individual to drug abuse or to a number of psychiatric diseases.	Yes
Burré et al. (2010)[17]	Brains from wild-type mice, CSPα -/- mice, and CSPα -/- rescued by transgenic α-synuclein and HEK293 cells.		Maintenance of continuous presynaptic SNARE-complex assembly required a non-classical chaperone activity mediated by synucleins.	Yes
Al-Wandi et al. (2010)[18]	2 year old α-synuclein -/- mice and γ-synuclein -/- mice.	Protein	The absence of this protein makes striatal dopaminergic synapses more vulnerable to changes associated with aging of the nervous system.	Yes
Greten-Harrison et al. (2010)	Neuronal cultures of wildtype and αβγ-synuclein -/- mice.	Protein	Synuclein deficiency leads to altered synapse structure and physiology, age-dependent neuronal dysfunction, and impaired survival.	Yes
Anwar et al. (2011)[19]	αβγ-synuclein -/- mice.	Protein	Synucleins in some dopaminergic neurons limit synaptic neurotransmission through mechanisms that differ from those reported in other neurons, which have included vesicle pool redistribution or SNARE complex formation.	Yes
Beatman et al. (2016)[20]	α-synuclein -/-, -/+, +/+ mice, neuronal cells from strial and parietal cortical tissue. Brain tissue from patients with West Nile virus infection.	Protein	Native α-synuclein expression in neurons inhibits viral growth and injury in the CNS.	Yes
Chen et al. (2018)[21]	α-Synuclein +/- and -/- cell lines of dopaminergic neurons from human stem cells.	mRNA, protein	Reducing or completely removing *SNCA* alleles by CRISPR/Cas9n-mediated gene editing confers a measure of resistance to Lewy pathology.	No

References

1. Neystat, M. *et al.* α-Synuclein Expression in Substantia Nigra and Cortex in Parkinson's Disease. *Mov. Disord.* **14**, 417–422 (1999).
2. Kholodilov, N. G. *et al.* Increased Expression of Rat Synuclein in the Substantia Nigra Pars Compacta Identified by mRNA Differential Display in a Model of Developmental Target Injury. *J. Neurochem.* **73**, 2586–2599 (1999).
3. Abeliovich, A. *et al.* Mice Lacking α-Synuclein Display Functional Deficits in the Nigrostriatal Dopamine System. *Cell Neuron* **25**, 239–252 (2000).
4. Alves da Costa, C., Ancolio, K. & Checler, F. Wild-type but Not Parkinson's Disease-related Ala-53 Thr Mutant α-Synuclein Protects Neuronal Cells from Apoptotic Stimuli. *J. Biol. Chem.* **275**, 24065–24069 (2000).
5. Alves, C., Paitel, E. & Vincent, B. α-Synuclein Lowers p53-dependent Apoptotic Response of Neuronal Cells. *J. Biol. Chem.* **277**, 50980–50984 (2002).
6. Lee, M., Hyun, D., Halliwell, B. & Jenner, P. Effect of the overexpression of wild-type or mutant Neuronal Cells-synuclein on cell susceptibility to insult. *J. Neurochem.* **76**, 998–1009 (2001).
7. Stefanis, L., Larsen, K. E., Rideout, H. J., Sulzer, D. & Greene, L. A. Expression of A53T Mutant But Not Wild-Type Neuronal Cells-Synuclein in PC12 Cells Induces Alterations of the Ubiquitin-Dependent Degradation System, Loss of Dopamine Release, and Autophagic Cell Death. *J. Neurosci.* **21**, 9549–9560 (2001).
8. Hashimoto, M. *et al.* Neuronal Cells-Synuclein Protects against Oxidative Stress via Inactivation of the c-Jun N-terminal Kinase Stress-signaling Pathway in Neuronal Cells. *J. Biol. Chem.* **277**, 11465–11472 (2002).
9. Seo, J. *et al.* α-Synuclein regulates neuronal survival via Bcl-2 family expression and PI3/Akt kinase pathway. *FASEB J.* **16**, (2002).
10. Cabin, D. E. *et al.* Synaptic Vesicle Depletion Correlates with Attenuated Synaptic α-Synuclein. *J. Neurosci.* **22**, 8797–8807 (2002).
11. Wersinger, C., Prou, D., Vernier, P. & Sidhu, A. Modulation of dopamine transporter function by α-synuclein is altered by impairment of cell adhesion and by induction of oxidative stress. *FASEB J.* **17**, (2003).
12. Zourlidou, A., Smith, M. D. P. & Latchman, D. S. Modulation of cell death by α-synuclein is stimulus-dependent in mammalian cells. *Neurosci. Lett.* **340**, 234–238 (2003).

13. Kingsbury, A. E. *et al.* Alteration in α-Synuclein mRNA Expression in Parkinson's Disease. *Mov. Disord.* **19**, 162–170 (2004).
14. Chandra, S. *et al.* Double-knockout mice for α- and β-synucleins: Effect on synaptic functions. *Proc. Natl. Acad. Sci. U. S. A.* **101**, 14966–14971 (2004).
15. Chandra, S., Gallardo, G., Fernández-chacón, R., Schlüter, O. M. & Südhof, T. C. α-Synuclein Cooperates with CSPα in Preventing Neurodegeneration. *Cell* **123**, 383–396 (2005).
16. Oksman, M., Tanila, H. & Yavich, L. Brain reward in the absence of alpha-synuclein. *Mol. Neurosci.* **17**, 1191–1194 (2006).
17. Burré, J., Sharma, M., Tsetsenis, T., Buchman, V. & Südhof, T. C. α-Synuclein Promotes SNARE-Complex Assembly in vivo and in vitro. *Science* **329**, 1663–1667 (2010).
18. Al-wandi, A. *et al.* Absence of α-synuclein affects dopamine metabolism and synaptic markers in the striatum of aging mice. *Neurobiol. Aging* **31**, 796–804 (2010).
19. Anwar, S. *et al.* Functional Alterations to the Nigrostriatal System in Mice Lacking All Three Members of the Synuclein Family. *J. Neurosci.* **31**, 7264–7274 (2011).
20. Beatman, E. L. *et al.* Alpha-Synuclein Expression Restricts RNA Viral Infections in the Brain. *J. Virol.* **90**, 2767–2782 (2016).
21. Chen, Y. *et al.* Engineering synucleinopathy-resistant human dopaminergic neurons by CRISPR-mediated deletion of the *SNCA* gene. *Eur. J. Neurosci.* **49**, 510–524 (2019).
