# Supplementary material for: Transcriptomic signatures of brain regional vulnerability to Parkinson’s disease
Source: Commun Biol. 2020 Mar 5;3:101. doi: 10.1038/s42003-020-0804-9 (PMC7058608; doi:10.1038/s42003-020-0804-9)
Supplement: Supplementary file 8 — Description of Additional Supplementary Files [file 42003_2020_804_MOESM8_ESM.pdf]

Supplementary Data 1 Braak stage related genes (BRGs). List of all BRGs with Braak correlations, fold-change between Braak region 1 and 6, and its Benjamini-Hochberg-corrected *P*-values.

Supplementary Data 2 Functional GO-terms associated with negative correlated BRGs.

Supplementary Data 3 Functional GO-terms associated with positive correlated BRGs.

Supplementary Data 4 Demographic and pathological data of postmortem brain tissue samples of controls, incidental Lewy body disease (iLBD), and Parkinson's disease (PD) cases in the PD microarray dataset.

Supplementary Data 5 Demographic and pathological data of postmortem brain tissue samples of controls and Parkinson's disease (PD) cases in the PD RNA-seq dataset.

Supplementary Data 6 Braak co-expression modules. Modules with the number of genes, eigengene correlation with Braak stages, Benjamini-Hochberg-corrected *P*-values of correlations, and genes within the modules.
