# Supplementary material for: Transcriptomic signatures of brain regional vulnerability to Parkinson’s disease
Source: Commun Biol. 2020 Mar 5;3:101. doi: 10.1038/s42003-020-0804-9 (PMC7058608; doi:10.1038/s42003-020-0804-9)
Supplement: Supplementary file 10 — Reporting Summary [file 42003_2020_804_MOESM10_ESM.pdf]

nature research

Corresponding author(s): Marie-Christine Chartier-Harlin, Wilma D.J. van de Berg, Jacobus J. van Hilten, Marcel J.T. Reinders

Last updated by author(s): Sep 2, 2019

Reporting Summary

Nature Research wishes to improve the reproducibility of the work that we publish. This form provides structure for consistency and transparency in reporting. For further information on Nature Research policies, see Authors & Referees and the Editorial Policy Checklist.

Statistics

For all statistical analyses, confirm that the following items are present in the figure legend, table legend, main text, or Methods section.

n/a	Confirmed	
☐	☒	The exact sample size (*n*) for each experimental group/condition, given as a discrete number and unit of measurement
☐	☒	A statement on whether measurements were taken from distinct samples or whether the same sample was measured repeatedly
☐	☒	The statistical test(s) used AND whether they are one- or two-sided<br>*Only common tests should be described solely by name; describe more complex techniques in the Methods section.*
☐	☒	A description of all covariates tested
☐	☒	A description of any assumptions or corrections, such as tests of normality and adjustment for multiple comparisons
☐	☒	A full description of the statistical parameters including central tendency (e.g. means) or other basic estimates (e.g. regression coefficient) AND variation (e.g. standard deviation) or associated estimates of uncertainty (e.g. confidence intervals)
☐	☒	For null hypothesis testing, the test statistic (e.g. *F*, *t*, *r*) with confidence intervals, effect sizes, degrees of freedom and *P* value noted<br>*Give P values as exact values whenever suitable.*
☒	☐	For Bayesian analysis, information on the choice of priors and Markov chain Monte Carlo settings
☐	☒	For hierarchical and complex designs, identification of the appropriate level for tests and full reporting of outcomes
☐	☒	Estimates of effect sizes (e.g. Cohen's *d*, Pearson's *r*), indicating how they were calculated

Our web collection on statistics for biologists contains articles on many of the points above.

Software and code

Policy information about availability of computer code

Data collection	For the Allen Human Brain Atlas, complete normalized microarray datasets in csv-format were downloaded from http://human.brain-map.org. Microarray data from the UK Brain Expression Consortium were downloaded from braineac.org in txt-format. For the Genotype-Tissue Expression project data, both gene read counts and gene TPMs RNA-Sequencing data v7 were downloaded from https://gtexportal.org/home/datasets in gct-format. Raw microarray data of the substantia nigra from Parkinson's disease, and incidental Lewy body disease patients, and controls are available at GEO (accession number GSE49036) in CEL-format. RNA-sequencing data from Parkinson's disease patients and controls were available in CPM-values.
Data analysis	Scripts were run in R version 3.5 and can be found online: https://github.com/arlinkeo/pd_braak. Scripts to analyze the microarray and RNA-seq datasets of PD patients were run in R version 3.4.

For manuscripts utilizing custom algorithms or software that are central to the research but not yet described in published literature, software must be made available to editors/reviewers. We strongly encourage code deposition in a community repository (e.g. GitHub). See the Nature Research guidelines for submitting code & software for further information.

Data

Policy information about availability of data

All manuscripts must include a data availability statement. This statement should provide the following information, where applicable:

- Accession codes, unique identifiers, or web links for publicly available datasets
- A list of figures that have associated raw data
- A description of any restrictions on data availability

Data from non-neurological subjects used in this study are publicly available at brain-map.org, braineac.org, and gtexportal.org. Microarray data from PD, and iLBD patients, and controls were collected and shared by Amsterdam University Medical Center, the Netherlands.

Field-specific reporting

Please select the one below that is the best fit for your research. If you are not sure, read the appropriate sections before making your selection.

☒ Life sciences ☐ Behavioural & social sciences ☐ Ecological, evolutionary & environmental sciences

For a reference copy of the document with all sections, see nature.com/documents/nr-reporting-summary-flat.pdf

Life sciences study design

All studies must disclose on these points even when the disclosure is negative.

Sample size	Sample sizes were chosen based on the available samples from each dataset given the regions of interest.
Data exclusions	Data samples were excluded when they do not correspond to the regions of interest.
Replication	Reproducibility was tested using other publicly available gene expression datasets from non-neurological brains (Genotype-Tissue Expression project and UK Brain Expression Consortium). Differential expression analysis could be replicated, however correlation analysis was not possible due to a limited sampling resolution compared to the Allen Human Brain Atlas.
Randomization	This does not apply to data from non-neurological subjects obtained from publicly available datasets. For the microarray and RNA-sequencing data from Parkinson's disease patients, all samples were analyzed in batches including patients and controls that were randomized.
Blinding	This does not apply to data from non-neurological subjects obtained from publicly available datasets. For the microarray and RNA-sequencing data from Parkinson's disease patients, brain tissue processing for microarray and RNA-sequencing was done by an experienced research technician who was blinded for all clinical and pathological data.

Reporting for specific materials, systems and methods

We require information from authors about some types of materials, experimental systems and methods used in many studies. Here, indicate whether each material, system or method listed is relevant to your study. If you are not sure if a list item applies to your research, read the appropriate section before selecting a response.

Materials & experimental systems

n/a	Involved in the study	
☒	☐	Antibodies
☒	☐	Eukaryotic cell lines
☒	☐	Palaeontology
☒	☐	Animals and other organisms
☐	☒	Human research participants
☒	☐	Clinical data

Methods

n/a	Involved in the study	
☒	☐	ChIP-seq
☒	☐	Flow cytometry
☒	☐	MRI-based neuroimaging

Human research participants

Policy information about studies involving human research participants

Population characteristics	In the Parkinson's disease microarray dataset, samples were collected from the medulla oblongata, locus ceruleus, and substantia nigra from Parkinson's disease patients (67.6% males, mean age 78, range 61–87 years), incidental Lewy body disease patients (42.4% males, mean age 80, range 56–98 years), and non-demented controls (54.5% males, mean age 77, range 60–91 years) (Supplementary Table 5 and 6). The data of the substantia nigra was previously published in Dijkstra et al. (2015). Based on pathological examination, Parkinson's disease patients revealed Lewy body pathology in accordance with Braak stages 4-6, and incidental Lewy body disease patients with Braak stages 1-3.<br>In the Parkinson's disease RNA-seq dataset, samples from the substantia nigra and medial temporal gyrus were collected from Parkinson's disease patients (61.1% males, mean age 79, range 57–88 years), and non-demented age-matched controls (48.0% males, mean age 78, range 59–93 years) (Supplementary Table 7 and 8).
Recruitment	Postmortem brain tissue was collected by the Netherlands Brain bank via donor registration program. See for more details: www.brainbank.nl
Ethics oversight	VU University Medical Center (VUmc, Amsterdam), Medical Ethics Committee (METC VUmc, reference number 2009/148), and 1964 Helsinki declaration.

Note that full information on the approval of the study protocol must also be provided in the manuscript.
